# *Piper aduncum* essential oil: a promising insecticide, acaricide and antiparasitic. A review

**DOI:** 10.1051/parasite/2021040

**Published:** 2021-05-03

**Authors:** Andrea Durofil, Matteo Radice, José Blanco-Salas, Trinidad Ruiz-Téllez

**Affiliations:** 1 Universidad Estatal Amazónica Km 2½ Vía Puyo-Tena 160150 Puyo Ecuador; 2 Department of Vegetal Biology, Ecology and Earth Science, Faculty of Sciences, University of Extremadura 06006 Badajoz Spain

**Keywords:** *Piper aduncum*, Essential oil, Insecticide, Acaricide, Antiparasitic, Dillapiole

## Abstract

Several studies have assessed the potential of essential oils as substitutes for synthetic pesticides, in order to counter insect resistance to commercial pesticides. *Piper aduncum* L. is a very common shrub in the Amazon Rainforest and in other subtropical areas. The objective of this review was to analyse the existing information on *P. aduncum* essential oil as a raw material for new bioproducts for sustainable pest disease management. With this review, we collected and critically analysed 59 papers, representing all the studies that aimed to evaluate the essential oil properties of this species as an insecticide, acaricide and antiparasitic. The chemical composition differs depending on the origin, although phenylpropanoid dillapiole is the most cited component, followed by myristicin, 1,8-cineole and β-ocimene. Between the acaricidal, antiparasitic and synergistic activities, the insecticidal effects are highly promising, with optimal results against the malaria vector *Aedes aegypti*, with an LC_50_ that ranges between 57 and 200μg/mL. Acaricidal activity has mainly been reported against *Tetranychus urticae*, showing an LC_50_ that ranges between 5.83 and 7.17μg/mL. Antiparasitic activity has predominately been found on *Leishmania amazonensis*, and antipromastigote activity has been found to be between 23.8 and 25.9μg/mL. Concerning the synergistic effect between dillapiole and synthetic insecticides, four studies on *Spodoptera frugiperda* found promising results with cypermethrin. In this review, we highlighted the potential of *P. aduncum* essential oil as a biopesticide, also focusing on the lack of information about applied research. We also provide suggestions for future investigations.

## Abstract (fr)

Plusieurs études ont abordé le potentiel des huiles essentielles comme substitut aux pesticides de synthèse, afin de lutter contre la résistance des insectes aux pesticides commerciaux. *Piper aduncum* L. est un arbuste très commun dans la forêt amazonienne et dans d’autres zones subtropicales. L’objectif de ce travail est d’analyser les informations existantes sur l’huile essentielle de *P. aduncum* comme matière première de nouveaux bioproduits pour une gestion durable des maladies nuisibles. Dans cette synthèse, nous avons collecté et analysé de manière critique 59 articles, représentant toutes les études qui visaient à prouver les propriétés des huiles essentielles de cette espèce en tant qu’insecticide, acaricide et antiparasitaire. La composition chimique diffère selon la provenance, bien que le phénylpropanoïde dillapiole soit le composant le plus cité, suivi de la myristicine, du 1,8-cinéole et du β-ocimène. À côté des activités acaricide, antiparasitaire et de synergie avec les autres composants, les effets insecticides sont les plus prometteurs, avec des résultats optimaux contre le vecteur du paludisme *Aedes aegypti*, avec une CL_50_ comprise entre 57 et 200μg/mL. Une activité acaricide a été principalement rapportée contre *Tetranychus urticae* avec une CL_50_ comprise entre 5,83 et 7,17μg/mL. L’activité antiparasitaire a été montrée principalement sur *Leishmania amazonensis* et l’activité antipromastigote s’est avérée être comprise entre 23,8 et 25,9μg/mL. Concernant l’effet synergique entre le dillapiole et les insecticides synthétiques, quatre études sur *Spodoptera frugiperda* ont mis l’accent sur des résultats prometteurs avec la cyperméthrine. Avec cette étude, nous avons mis en évidence le potentiel de l’huile essentielle de *P. aduncum* en tant que biopesticide, en insistant sur le manque d’informations de la recherche appliquée et sur certaines propositions pour de futures recherches.

## Introduction

About 80% of the world population relies predominantly on plants and plant extracts for health care [81]. Starting from the Orient, mainly from Egypt, Persia and India, through to the Western World, from the ancient Greeks to the Romans, and finding a clear definition in the Middle Ages, Essentials Oils (EOs) are a fundamental part of the history of our civilization [37].

After World War II, there was new trend to mostly study synthetic chemical substances, which led to a decrease in the use of botanical extracts. Clearly, synthetic chemical compounds are more effective compared to natural extracts, but the lack of knowledge about natural substances is too great to compare them fairly: only a small fraction of the 250,000 plant species has been studied properly [91, 102].

Every day, there is increasing evidence about the consequences of using synthetic antiparasitics and insecticides, including their role in environmental pollution, their residual presence in foods and feeds, and most of all their function in developing resistance in parasites and insects [10, 33, 39–41, 85].

The Amazon Rain Forest is one of the most megadiverse places in the world, with more than 50,000 plant species, including at least 14,000 seed plants [13]. For instance, more than 250 species are used by indigenous communities as medicinal treatments in just a small corner of the forest [101].

For the same reason, there are thousands of studies that aim to define the proprieties of the EOs derived from this megadiversity, such as *Viola surinamensis* [57], *Guatteriopsis* species [20] and *Lippia grandis* [89].

*Piper* species belong to one of the largest genera of basal angiosperm [97] and are widespread in the tropical and subtropical regions of the world. They have a long history of use in traditional medicine and many studies have aimed to prove their efficacy. For instance, EOs from *P. auritum* showed promising results in the inhibition of promastigote proliferation in different species of the *Leishmania* genus [67], and EOs from *P. hispidum* showed high antileishmanial activity [43]. Also, *P. cubeba* EOs have anti-trypomastigote and anti-amastigote activity in *Trypanosoma cruzi* [24]. Various compounds from different species have been studied to assess these activities: dihydrochalcones from *P. longicaudatum,* where asebogenin (2′,6′,4-trihydroxy-4′-methoxydihydrochalcone) showed inhibitory effects against *Staphylococcus aureus* and methicillin-resistant *S. aureus* (MRSA) [48]; prenylated hydroquinone from *P. crassinervium* with trypanocidal activity [56]; and eupomatenoid-5 isolated from leaves of *P. regnellii* which induces apoptosis in *Leishmania amazonensis* [36].

**Figure 1:**
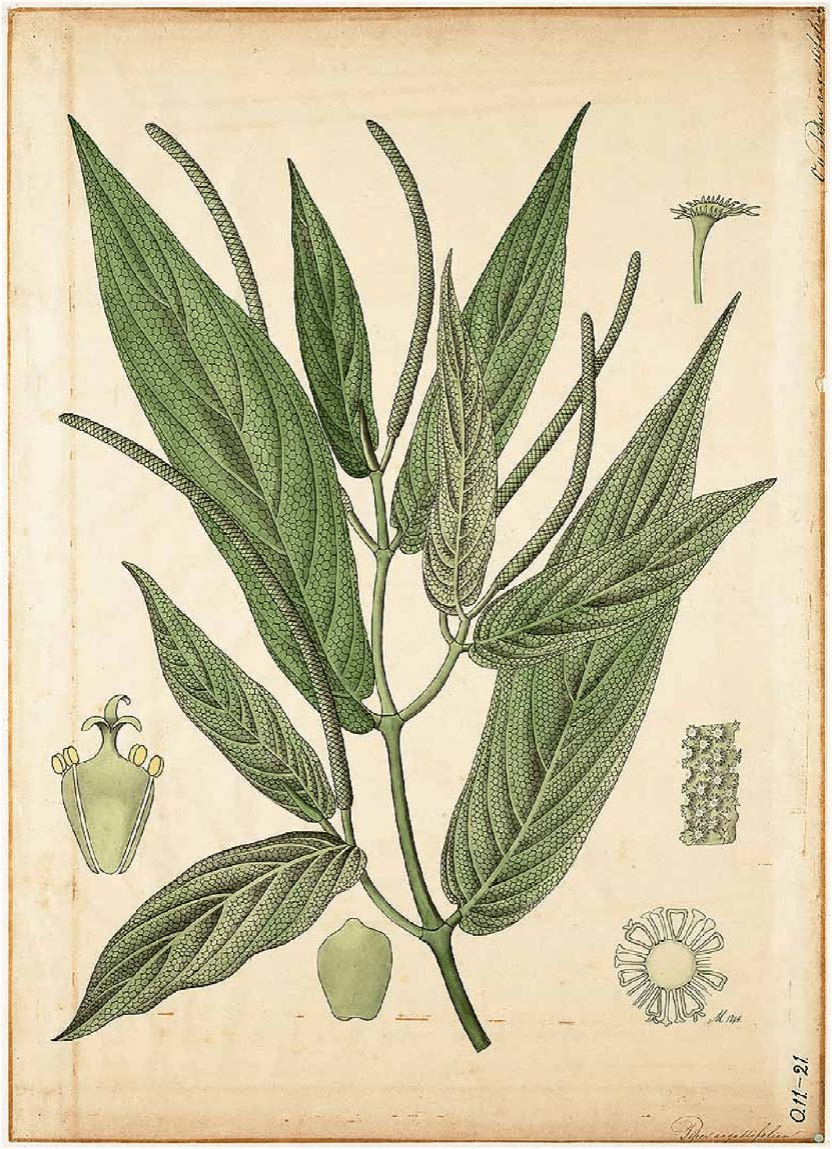
*Piper aduncum* L. (from Botanischen Wandplatet at http://www.plantillustrations.org/.

In this genus, we also find *P. aduncum,* which has a geographic range that extends mainly through the Neotropics, Southern Asia and the South Pacific [47]. It is a shrub or small tree up to 2–5 (−8) m tall with pubescent stems. Leaves up to 20cm long and 5–9 wide, oblong-elliptic or lanceolate, scabrous on the upper surface and pubescent underneath, acuminate, base rounded or slightly lobed, alternate, distichous. Short petioles rarely up to 8cm, pubescent. Prophylls up to 25mm present. Inflorescence 5–17cm, forming arching cream to green spikes on peduncles 8–15mm long, sparsely pubescent. Floral perianth absent. Androecium with four stamens, anthers 0.2–0.3mm long. Floral bracts 0.4–0.7mm wide, triangular-round, densely yellow-white ciliate. Ovary with three stigmas. One-seeded berries 0.8–1mm wide, obovoid, round from above, glabrous. Seeds reticulated [100] (Fig. 1).

Several ethnobotanical uses have been reported, such as the treatment of inflammation and cuts, skin irritations, “bone pains” and nasal haemorrhage. A tea made from its leaves is used to stop pulmonary haemorrhage, to treat tenesmus in women in labor, or to relieve menstrual colic. It is also mentioned as a folk remedy against kidney disorders, stomach ache, ulcers, rheumatism and skin infections; it is externally applied to relieve skin eruptions in babies. This same tea was described as a diuretic when mixed with corn silk tea. The ethanolic extract obtained from dry leaves was traditionally used to relieve headache and water infusion of buds to lower cholesterol. The entire plant has been mentioned for hot baths in order to help patients during their convalescence; moreover, the tea has been described as a traditional antihemorrhagic and a health tonic [6, 22].

De Castro *et al.* provided evidence of the effectiveness of cardamonin as a schistosomicidal chalcone from *P. aduncum* extracts, inhibiting the ATP diphosphohydrolase of *Schistosoma mansoni*, the major aetiological agent of human schistosomiasis [14].

Many of the biological proprieties of *P. aduncum* EO have been studied, for instance its antibacterial and antifungal activity [68], which showed good results against the problematic agents of nosocomial infections such as *Staphylococcus aureus, S. epidermidis* and *S. lentus* [9]. Similarly, there are promising results in the prevention of infection in immunocompetent or immunocompromised patients for its activity against *Cryptococcus neoformans* [69]. There are also antioxidant [45], anti-inflammatory [74] and antiplatelet [38] activities, among others.

In order to carry out a systematic review, we collected all the studies regarding insecticidal, acaricidal and antiparasitic activity, mostly aiming to find a way to describe results which can be useful for an alternative or complementary strategy against leishmaniasis and malaria, considering that these diseases are still a great challenge in many countries from both the Eastern and Western tropical and subtropical regions [51].

## Materials and methods

To assess the compound’s activities, we selected articles from the electronic databases PubMed (https://pubmed.ncbi.nlm.nih.gov/), SciFinder (https://scifinder.cas.org), ScienceDirect (https://www.sciencedirect.com/), ISI-Web of Science (http://apps.webofknowledge.com), SciELO (https://scielo.org/) and Google Scholar (https://scholar.google.com/). To manage bibliographic references, we used Mendeley software (https://www.mendeley.com/). Most of the papers date from the last 20years, but we also included some key data starting from 1948 to develop the introduction. To gather information, we only considered those articles regarding the use of the essential oil in its totality or the compounds derived from it: no semi-synthetic element’s activity is shown in this review.

We collected all articles related, on the one hand to antiparasitic activity mainly focusing on the *Leishmania* genus, and on the other, to insecticide properties, most of all regarding the multiple studies on controlling malaria vectors, among others. And finally, we collected articles on acaricidal activity and the applications of this volatile oil in synergy with already known chemical insecticides.

We chose “essential oil”, “insecticide”, “acaricide”, “antiparasitic” and “dillapiole” as keywords and searched them in different combinations with the main keyword “*Piper aduncum*”.

To form the tables, we selected the following criteria: geographic distribution of the plant used for the extraction, parts of the plant used, method of extraction, main compounds found in the oil, type of application, and organism in which it was applied and activity that it produced in that organism, specifying the concentration used to obtain different ranges of the same activity (when not specified, we reported the effect of the minor time experiments).

## Results and discussion

### Geography and aim of the studies

**Figure 2:**
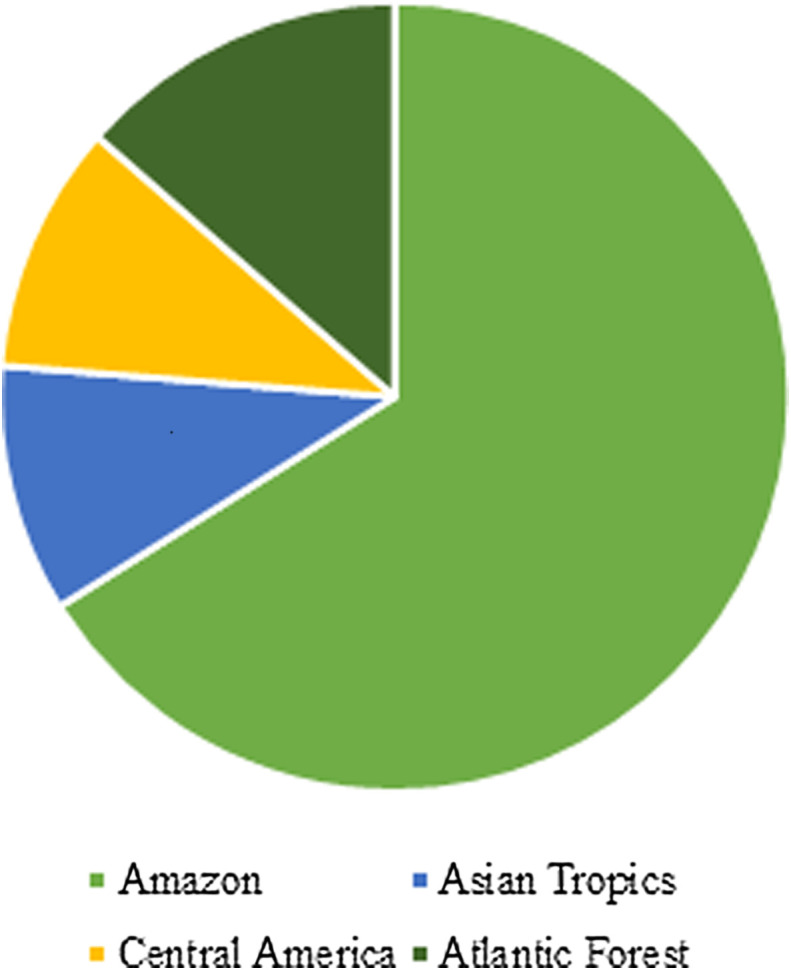
Biogeographic precedence of plant material in the reviewed papers.

Using these criteria, we were able to collect 59 papers, in English, Portuguese and Spanish. As shown in Figure 2, most of the articles aimed to analyse the properties of the EOs of *P. aduncum* plants taken from the Amazon Rain Forest. We counted 39 from the Amazon, whereas only 8 were from the Atlantic Forest, and just 6 from both the Central America region (Cuba) and the Asian Tropics (Malaysia).

**Figure 3:**
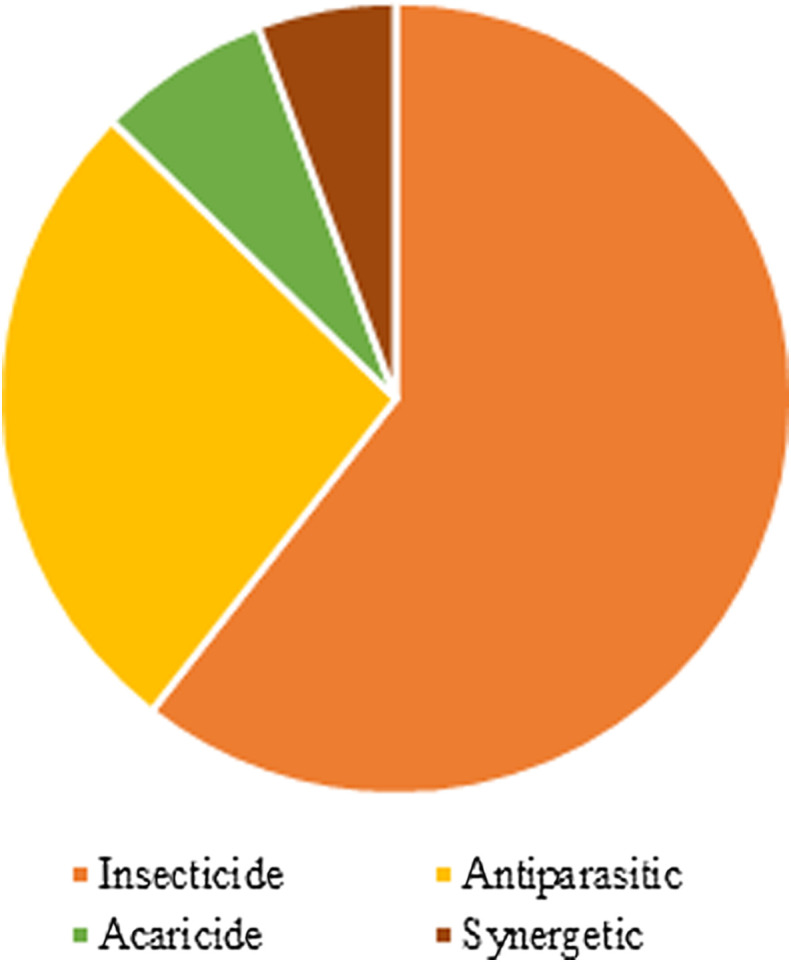
Distribution of the focus of the topics in the revised bibliography.

Most of these articles focused on insecticidal activity: we considered 43 experiments (60%) which aimed to determine this type of activity. We also counted 5, 19 and 4 experiments (7, 27 and 6%) regarding the acaricidal and antiparasitic activities and the synergistic effect as an insecticide, respectively (Fig. 3).

### Extraction of the EO and its components

To obtain the essential oil, the leaves were the part of the plant mostly used in the experiments, sometimes together with fine stems. Regarding extraction methods, hydro-distillation (HD) by the conventional method using a Clevenger type apparatus was the most common, except in the experiments that defined the synergistic effect of the EO, which used steam-distillation (SD) to extract the oil. Gas chromatography was the basic procedure used, mainly together with mass spectrometry, to analyse and determine its chemical properties.

### Compounds of the essential oil

The chemical composition of the EO in the different studies mainly shows two large groups: phenylpropanoids and monoterpenes (Table 1).

**Table 1:** Chemical structure of the main components of *P. aduncum* essential oil. From the PubChem database (https://pubchem.ncbi.nlm.nih.gov/).

	Structure	Name	IUPAC Name
Phenylpropanoids	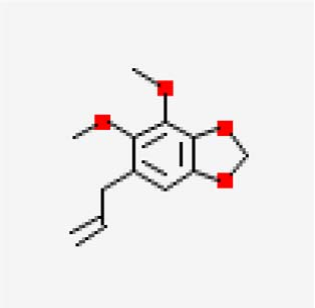	Dillapiole	4,5-dimethoxy-6-prop-2-enyl-1,3-benzodioxole
	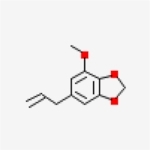	Myristicin	4-methoxy-6-prop-2-enyl-1,3-benzodioxole
	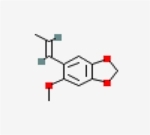	Carpacin	5-methoxy-6-[(E)-prop-1-enyl]-1,3-benzodioxole
	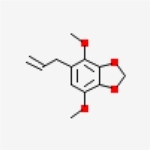	Apiole	4,7-dimethoxy-5-prop-2-enyl-1,3-benzodioxole
	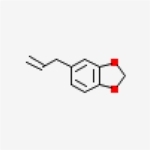	Safrole	5-prop-2-enyl-1,3-benzodioxole
	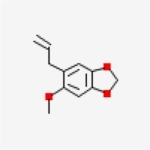	Sarisan	5-methoxy-6-prop-2-enyl-1,3-benzodioxole
Monoterpenes	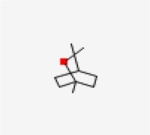	1,8-cineole	1,3,3-trimethyl-2-oxabicyclo[2.2.2]octane
	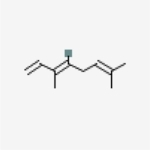	β-ocimene	(3E)-3,7-dimethylocta-1,3,6-triene
	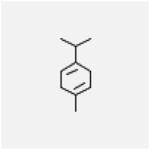	γ-terpinene	1-methyl-4-propan-2-ylcyclohexa-1,4-diene

According to Salehi *et al.* (2019) [84], the *Piper* genus is known to be a source of essential oils and *Piper* plants may contain different EOs from several organs and tissues such as seeds, leaves, fruits, branches, stems, and roots; more than 270 identified compounds have been found in *Piper* species. However, phytochemical studies on essential oils of different *Piper* species have pointed out high variability in chemical composition. As mentioned by Silva *et al*. (2017) [93], studies regarding essential oil composition of different *Piper* species found nine chemotypes characterised by 1,8-cineole, (E)-nerolidol, dillapiole and asaricin. Additionally, due to the resilient nature of the plants, the direct consequence of this biological approach is that the variability of secondary metabolism reflects the adaptation capacity of the plant to all external factors [16, 61]. This chemical evidence concerning essential oil variability is highly relevant to identify specific chemotypes that could be useful in the modern food and health products market, even though variable composition is a frequent obstacle to their use. The present review, pointing out the main constituents reported by different research, sheds light on these aspects of variability within the species.

Dillapiole was almost always the main component characterising the oil, followed by myristicin. However, the articles highlight variable abundance mainly due to the different growth conditions and geographical origins, which inevitably affect the qualitative and quantitative profile of the phytocomplex. Dillapiole was the most cited component and the one that had the most promising proprieties, but importantly dillapiole showed better activities as a component of the entire EO than as an isolated compound. Due to this variability, dillapiole amounts ranged from 9.20% [94] to 94.84% [87], even though several studies reported that dillapiole represents more than the 70% of total essential oil composition, as reported in Tables 2–5.

**Table 2:** Insecticidal activity.

Country	Part	Extraction	Main compound	Application	Insect	Activity	Ref
Brazil	NS	NS	NS	Contact (contaminated surface: filter paper and grain)	*Sitophilus zeamais* (Maize weevil) (Coleoptera)	Adulticide (filter paper: LC_50_=0.6μL/cm^2^, LC_95_=1.38μL/cm^2^; grain: LC_50_=0.64μL/g, LC_95_=12.74μL/g)	[59]
Brazil, Acre (Embrapa Acre)	Aerial parts	SD	Dillapiole (73.97%), safrole (3.92%), sarisan (2.84%)	Fumigation; Contact; Topical	*Sitophilus zeamais* (Maize weevil) (Coleoptera)	Adulticide (fumigation: LC_50_=0.56μL/g; contact: LC_50_=2.87μL/cm^2^; topical: LD_50_=0.03μL/g of insect)	[104]
Brazil, Acre (Embrapa Acre)	Aerial parts	SD	Dillapiole (69.3, 79.9 and 85.4%)	Topical	*Diaphorina citri* (Asian citrus psyllid) (Hemipteran)	Nymphicide (dillapiole 69.3%=95.71% mortality; 79.9%=97.14%; 85.4%=98.57%)	[106]
				Residual contact		Adulticide (69.3%=46.25%; 79.9%=88.75%; 85.4%=96.25%)	
Brazil, Acre (Embrapa Acre)	Aerial parts	SD	Dillapiole, safrole, sarisan	Topical; Contact (contaminated surface)	*Cerotoma tingomarianus* (Coleoptera)	Adulticide (topical: LD_50_=0.002 mL/mg of insect; contact: LC_50_=0.06 mL/cm^2^)	[26]
Brazil, Acre (Embrapa Acre)	Leaves	HD	NS	Fumigation;	*Callosobruchus maculatus* (Cowpea weevil) (Coleoptera)	Adulticide (LC_50_=169.50μL/L air); Repellence to oviposition (0.5 mL/kg reduced 66.06%)	[72]
				Repellent		Ovicide (68.63% at 0.5 mL/kg); Repellence (attraction reduced±5%)	
Brazil, Acre (Embrapa Acre)	Leaves	HD	Dillapiole (73.97%)	Topical; Contact (contaminated surface)	*Tenebrio molitor* (Mealworm) (Coleoptera)	Larvicide (topical: LD_50_=0.009μL/mg of insect; contact: LC_50_=0.033μL/cm^2^)	[27]
Brazil, Acre (Embrapa Acre)	Leaves	SD	Dillapiole (71.9%)	Topical and residual contact	*Spodoptera frugiperda* (Fall armyworm) (Lepidoptera)	Larvicide (topical: LD_50_=1.07μL/mg of insect; residual: LC_50_=1169.70ppm)	[30]
Brazil, Acre (Embrapa Acre)	Leaves	SD	Dillapiole (71.9%)	Topical and residual contact	*Spodoptera frugiperda* (Fall armyworm) (Lepidoptera)	Larvicide (topical: LD_50_=0.012μL/mg of insect; residual: LD_50_=0.00011μL/cm^2^)	[29]
Brazil, Acre (Embrapa Acre)	Leaves	SD	Dillapiole (71.9%)	Topical and residual contact	*Spodoptera frugiperda* (Fall armyworm) (Lepidoptera)	Larvicide (topical: LD_50_=0.00011μL/insect; residual: LC_50_=1169.7ppm)	[28]
Brazil, Acre (Embrapa Acre)	Leaves	SD	Dillapiole (71.9%)	Topical and residual contact	*Spodoptera frugiperda* (Fall armyworm) (Lepidoptera)	Larvicide (topical: LD_50_=0.00011μL/mg of insect; residual: LC_50_=0.012μL/cm^2^)	[31]
Brazil, Acre (Embrapa Acre)	NS	NS	Dillapiole (73.97%), safrole (3.92%), sarisan (2.84%)	Contact	*Callosobruchus maculatus* (Cowpea weevil) (Coleoptera)	Adulticide (100%=50μL/20g)	[77]
						Ovicide (>90%=50μL/20g); Blocking egg-hatching (>90%=50μL/20g)	
Brazil, Amazonas	Leaves	HD	*Dillapiole	Contact (dilution in water)	*Aedes aegypti* (Yellow fever mosquito) (Diptera)	Larvicide (LC_50_=200μg/mL)	[79]
						Pupaecide (LC_50_=200μg/mL)	
Brazil, Amazonas (INPA)	Leaves	HD	*Dillapiole	Contact	*Drosophila melanogaster* (Fruit fly) (Diptera)	Larvicide (400μg/mL=70%, 2000μg/mL=100%)	[1]
Brazil, Amazonas	Leaves	HD	Dillapiole (52.37%), γ-terpinene (8.98%)	Contact	*Cerataphis lataniae* (Palm aphid) (Hemiptera)	Adulticide (LC_50_=219.4μg/mL, LC_90_=864.8μg/mL; loaded nanoparticles 500μg/mL=90%)	[94]
				Contact (gelatine nanoparticles)			
Brazil, Amazonas	Leaves	HD	Dillapiole (52.37%), γ-terpinene (8.98%)	Contact; Contact (gelatine nanoparticles)	*Aedes aegypti* (Yellow fever mosquito) (Diptera)	Larvicide (LC_50_=68.2μg/mL, LC_90_=125.3μg/mL; loaded nanoparticles 500μg/mL=100%)	[94]
Brazil, Amazonas	Leaves	SD	*Dillapiole	Contact	*Aedes aegypti* (Yellow fever mosquito) (Diptera)	Adulticide (LC_50_=0.381μL/cm^2^, LC_90_=0.575μL/cm^2^)	[96]
Brazil, Mato Groso	Leaves	HD	Myristicin (30.03%), aromadendrene (9.20%), dillapiole (8.43%), α-serinene (7.31%), tridecane (6.26%), γ-elemene (4.58%), o-cymene (4.20%)	Topical	*Euschistus heros* (Brown stink bug) (Hemiptera)	Adulticide (LD_50_=36.23mg; LD_90_=50.42mg)	[19]
Brazil, Mato Groso	Leaves	HD	Myristicin, isomyristicin, asaricim, dillapiole, isocroweacin	Ingestion; Topical	*Helicoverpa armigera* (Cotton bollworm) (Lepidoptera)	Larvicide (ingestion: 1^st^ instar LT_50_=<14.20days, 3^rd^ LT_50_=<16.89; topical: 1^st^ LT_50_=<14.68, 3^rd^ LT_50_=<10.73)	[88]
Brazil, Mato Groso	Leaves	HD	Myristicin (30.03%), aromadendrene (9.20%), dillapiole (8.43%), α-serine (7.31%), tridecane (6.26%), γ-elemene (4.58), o-cymene (4.20%)	Contact and Immersion (eggs); Topical	*Euschistus heros* (Brown stink bug) (Hemiptera)	Ovicide (Immersion: LC_50_=15.64mg/mL; Contact: LC_50_=24.29mg/mL)	[103]
						Nymphicide (LD_50_=11.37mg/mL; LD_90_=38.95mg/mL)	
						Adulticide (>20mg/mL: LT_50_=<6 days)	
Brazil, Mato Groso	Leaves	SD	Dillapiole, z-carpacin, myristicin	Topical	*Tibraca limbativentris* (Stink bug) (Hemiptera)	Ovicide (LC_50_=2.499%)	[49]
Brazil, Mato Groso	Leaves	SD	Dillapiole, myristicin, z-carpacin	Topical; Ingestion	*Chrysodeixis includens* (Soybean looper) (Lepidoptera)	Larvicide (at 24h: ingestion: LC_50_=3.5%, LC_90_=6.2%; topical: LC_50_=16.2% LC_90_=30.0%)	[86]
Brazil, Minas Gerais	Leaves	HD	1,8-cineole (53.9%), α-pinene (12.7%), β-pinene (8.5%), trans-ocimene (5.7%)	Fumigation (dilution in water)	*Aedes aegypti* (Yellow fever mosquito) (Diptera)	Larvicide (250ppm=40%, 500ppm=100%)	[70]
Brazil, Pará	Aerial parts	HD	Dillapiole (64. 4%)	Contact (contaminated surface)	*Solenopsis saevissima* (Fire ant) (Hymenoptera)	Adulticide (LC_50_= 58.4mg/L)	[99]
Brazil, Pará	Leaves	HD	Asaricine, myristicin, dillapiole, (E)-β-ocimene, piperitone	Spraying	*Anticarsia gemmatalis* (Velvetbean caterpillar) (Lepidoptera)	Ovicidal (LC_50_=1.9%, LC_90_=2.8%)	[50]
Brazil, Pará	NS	NS	Dillapiole (73.97%), safrole (3.92%), sarisan (2.84%)	Topical	*Callosobruchus maculatus* (Cowpea weevil) (Coleoptera)	Ovicide (100%=0.5 L/t); Block egg-hatching (100%=0.5l/t)	[76]
Brazil, Paraiba	Dried fruit	HD	β-pinene (32.7%), E-caryophyllene (17.1%)	Contact (dilution in water)	*Aedes aegypti* (Yellow fever mosquito) (Diptera)	Larvicide (LC_50_=30.2μg/mL)	[21]
Brazil, Rondonia	Leaves	HD	(E)-isocroweacin (29.52%) apiole (28.62%) elemicin (7.82%)	Fumigation (dilution in water)	*Aedes aegypti* (Yellow fever mosquito) (Diptera)	Larvicide (LC_50_=46ppm; LC_90_=156ppm; 100%=500ppm)	[87]
Colombia, Chocó	Leaves	HD	Dillapiole (48,2%), 1,8 cineole (11,4%)	Contact (contaminated surface)	*Triblium castaneum* (Red flour beetle) (Coleoptera)	Repellence (1μL/cm^2^= 99% for 2h)	[46]
Cuba, La Habana	Leaves	HD	Dillapiole (82.0%)	Fumigation (dilution in water)	*Aedes aegypti* (Yellow fever mosquito) (Diptera)	Larvicide (LC_50_=57mg/L; LC_90_5=75mg/L)	[52]
Cuba, La Habana	NS	SD	NS	Fumigation (dilution in water)	*Aedes aegypti* (Yellow fever mosquito) (Diptera)	Larvicide (LC_50_=36.0mg/mL)	[60]
						Adulticide (60mg/mL LT_50_=0.19h)	
Cuba, La Habana	NS	SD	NS	Fumigation (dilution in water)	*Aedes aegypti* (Yellow fever mosquito) (Diptera)	Larvicide (LC_50_=35.3mg/mL)	[60]
						Adulticide (30mg/mL LT_50_=0.15h)	
Cuba, La Habana	NS	SD	NS	Fumigation (dilution in water)	*Aedes aegypti* (Yellow fever mosquito) (Diptera)	Larvicide (LC_50_=57.3mg/mL)	[60]
						Adulticide (40mg/mL LT_50_=0.19h)	
Cuba, La Habana	NS	SD	NS	Fumigation (dilution in water)	*Culex quinquefasciatus* (Southern house mosquito) (Diptera)	Larvicide (LC_50_=59.5mg/mL)	[60]
						Adulticide (60mg/mLLT_50_= 0.17h)	
Cuba, La Palma	NS	HD	NS	Topical	*Musca domestica* (Housefly) (Diptera)	Adulticide (LC_50_=0.04%; LC_95_=0.33%)	[53]
Ecuador, Pastaza,	Aerial parts	HD	Dillapiole (48.2%), trans-ocimene (7.5%), β-caryophyllene (17.0%)	Fumigation (dilution in water)	*Aedes aegypti* (Yellow fever mosquito) (Diptera)	Larvicide (LC_50_= 23.73ppm; LC_90_= 35.51ppm; LC_99_= 49.31ppm)	[90]
Malaysia	NS	HD	(E)-β-ocimene, trans- caryophyllene, (z)-β- ocimene, β-pinene, α-pinene, germacrena-D, piperitone, γ-terpinene, limonene	Contact	*Periplaneta americana* (American cockroach) (Blattodea)	Adulticide (80,000ppm; females LC_50_=5.31h, LT_90_=14.9h, males LT_50_=2.08h, LT_90_=5.14h)	[54]
						Nymphicide (80,000ppm, LT_50_=4.68h, LT_90_=28.71)	
Malaysia, Selangor	Leaves	HD ***	apiole (38.01%), methyl isobutyl ketone (8.26%), piperitone (3.34%), caryophyllene (2.45%)	Topical (on human body)	*Aedes aegypti* (Yellow fever mosquito) (Diptera)	Repellence to human bodies (>65% at 4h post-application)	[58]
Malaysia, Selangor	Leaves	HD	NS	Spraying	*Aedes aegypti* (Yellow fever mosquito) (Diptera)	Adulticide (LC_50_=5.6%; LC_90_=12.3%)	[63]
Malaysia, Selangor	Leaves	HD	NS	Spraying	*Aedes aegypti* (Yellow fever mosquito) (Diptera)	Adulticide (LC_50_=5.5%; LC_90_=12.7%)	[63]
Malaysia, Selangor	Leaves	HD **	NS	Topical (human body)	*Aedes aegypti* (Yellow fever mosquito) (Diptera)	Repellence to human bodies (ED_50_=0.4%; ED_90_5=1.7%)	[42]
Malaysia, Selangor	Leaves	HD	NS	Topical (human body)	*Aedes aegypti* (Yellow fever mosquito) (Diptera)	Repellence to human bodies (60″ exposure: ED=1.5g/cm^2^)	[65]
Malaysia, Selangor	NS	HD	NS	Topical (human body)	*Aedes aegypti* (Yellow fever mosquito) (Diptera)	Repellence to human bodies (90″ exposure: ED_50_=1.95μg/cm^2^; ED_90_=18.1μg/cm^2^)	[64]
NS	NS	SD	*Dillapiole	Contact; Residual contact	*Leptinotarsa decemlineata* (Colorado potato beetle) (Coleoptera)	Larvicide (0.1ppm=92%)	[55]

* Only the main compound was tested;

** Ointment, cream and gel;

*** Dried over anhydrous magnesium sulphate then formulated into Carbopol 934 hydrogels, Aerial parts: Leaves and Branches, HD: hydro-distillation, SD: steam-distillation, NS: not specified, EO: essential oil, LC_50_: lethal concentration for 50%, LC_90_: lethal concentration for 90%, LD_50_: lethal dose for 50%, LD_90_: lethal dose for 90%, LT_50_: lethal time for 50%, LT_90_: lethal time for 90%, ED_50_: effective dose for 50%, ED_90_: effective dose for 90%, ED_95_: effective dose for 95%.

For EOs, variable composition is a frequent condition that makes it difficult to develop standardised batches. The choice and selection of specific chemotypes, the study of the balsamic period of the species and rigorous quality control on extraction, can partially mitigate the problem of variability, allowing production with reproducible quantities of the main components.

Like other phenylpropanoids, dillapiole is a result of the shikimic acid pathway [11] and several authors found biological activities of the isolated molecule or EOs rich in dillapiole. It can be found in a variety of plants, and various studies have aimed to define its uses or functions. These include its gastroprotective function when extracted from *Peperomia pellucida* [82], and its toxicity against the fungus *Leucoagaricus gongylophorus* in the control of fungus-feeding ants (Tribe Attini), an agricultural pest in the Neotropics [83]. It is a phenylpropanoid constituted by a benzodioxole with a methyl group in the aromatic ring and an alkyl group in the side chain. The structure of this organic compound is strictly related to its function and activities [74], and when we take a wider view on its synergistic function with some other compounds (Table 1), we observe that this same structure helps to amplify the effects of these chemical substances. This supports its potential when mixed, and not isolated. Finally, other articles cite the activity of semisynthetic derivatives of dillapiole against certain species of the genus *Aedes* [23, 34], and show its cytotoxic effects against a variety of tumour cells [32].

### Insecticidal activity

In Table 2, we show the results obtained in experiments on the insecticidal properties of the EO. We indicate the scientific names and how the insect is commonly classified. When specified, the concentration of dillapiole ranged between the 8.43% to the 85.4% if the EO was used in its totality. Only four experiments did not show the presence of dillapiole: one of them used dried fruits to extract the EO, differing from most of the other experiments that mainly used leaves. Many papers focused on activity against *Aedes aegypti*, a mosquito vector of several viruses.

Concerning use of the EO, or its main compound in these experiments that isolated it from the rest of the components, a variety of methods were used: topical, with direct application to the insect using a specialized instrument regarding the type or stage of the insect; contact, allowing the insect to move through a surface where the EO was placed, or through the substance diluted in water; fumigation, mostly obtained by evaporation of the solution containing diluted EO; immersion, where the organism was directly submerged in the solution; and spraying. For the fumigation and contact experiments, residual effects were also often determined. Finally, activity was expressed in multiple forms, for instance adulticide, larvicide and ovicide effects, or repellency to oviposition. Almost all results expressed the lethal concentration (LC) of the EO that affected at least 50% of the insects tested. This concentration was also expressed as lethal dose (LD), lethal time (LT) or effective dose (ED). ED was related to the efficiency of repelling mosquitos from the human body. These measurements depended on how the researcher wished to express EO lethality. Considering for example the larvicidal effect in *A. aegypti* and where dillapiole was the main compound of the EO applied through contact or fumigation, we find that the LC_50_ ranged between 57 and 200μg/mL.

Finally, when not specified, we reported only experiments with a lowest duration as there was no significant difference between them: generally, the longer the experiment duration, the lower the concentration needed to obtain the same efficiency.

*Piper aduncum* EO appears to have promising properties in terms of insecticidal activity as it has been tested in a wide range of different insects and provided positive results. On the other hand, there are many studies that report this property for others EOs, for instance recently described activity of *Foeniculum vulgare* EO extracts against certain aphid species [75]. These findings support the possibility of discovering suitable substitutes for chemical insecticides.

### Acaricidal activity

Table 3 shows the properties of the EO as an acaricidal and, like in Table 1, we report both classification and scientific name. We were able to collect only five experiments, three of them regarding the effects against *Tetranychus urticae*, a species of plant-feeding mite that is considered as a pest.

**Table 3:** Acaricidal activity.

Country	Part(s)	Extraction	Main Compound(s)	Application(s)	Mite	Activity(ies)	Ref
Brazil, Amazonas (Ducke Reserve)	Leaves	Hexane extract+HD	Dillapiole (94.84%)	Immersion	Tick (*Rhipicephalus microplus*)	Larvicide (0.1mg/mL=100%)	[92]
Brazil, Amazonas	Leaves	HD	Dillapiole (52.37%), γ-terpinene (8.98%)	Contact Contact (gelatine nanoparticles)	Mite (*Tetranychus urticae*)	Adulticide (LC_50_=56.5μg/mL, LC_90_=84.3μg/mL; loaded nanoparticles 500μg/mL=100%)	[94]
Brazil, Pernambuco	Leaves	HD	Dillapiole (76.5%)	Fumigation Residual contact	Mite (*Tetranychus urticae*)	Adulticide (fumigation: LC_50_=0.008μl/L air; residual: LC_50_=5.83μL/mL) Repellence to oviposition (fumigation: 0.001μL/L air=40%; residual: 0.0001μL/mL=30%)	[4]
Brazil, Pernambuco	Leaves	HD	Dillapiole (28%), α-humulene (1.6%), (E)-nerolidol (0.07%), β-caryophyllene (0.21%)	Fumigation Contact	Mite (*Tetranychus urticae*)	Adulticide (Fumigation LC_50_=0.01μL/L air; Contact LC_50_=7.17μL/mL) Repellence (RC_50_=0.04μL/mL)	[3]
Cuba, La Habana	Leaves	HD	Camphene, camphor, piperitone, viridiflorol	Contact	Mite (*Varroa destructor*)	Adulticide (25μL/Petri dish=100%)	[78]
HD: hydro-distillation, LC_50_: lethal concentration for 50%, LC_90_: lethal concentration for 90%, RC_50_: repellent concentration for 50%.							

The concentration of dillapiole ranged between 94.84% and 28%, and in just one of the experiments using plants of *P. aduncum* from Cuba, dillapiole was not present as a component of the EO. The application methods were similar to those used in the insecticide experiments.

There are fewer data on the acaricidal effects, but they still show promise regarding the control of pests in a wide variety of crops and cattle. In this field, we also found many studies on the use of the EOs as acaricides, for instance extracts of *Lippia gracilis* against *Tetranychus urticae* [8] or *Ocimum gratissimum* against *Rhipicephalus microplus* [95].

### Antiparasitic activity

In Table 4, we report antiparasitic activities, specifying the scientific name of each organism. Articles have been published concerning the toxicity of the EO against various species, such as *Haemonchus contortus* and *Hysterothylacium* sp., whereas most of the experiments aimed to determine activity against the genus *Leishmania*, the protozoa responsible for leishmaniasis, followed by *Trypanosoma*, which causes Chagas disease. Lastly, a number of studies were related to *Plasmodium falciparum*, suggesting that it may be more likely to overcome malaria using essential oils against its vector (Table 2), rather than approaches against the protozoa that causes the disease.

**Table 4:** Antiparasitic activity.

Country	Part(s)	Extraction	Main compound(s)	Application(s)	Parasite	Activity(ies)	Ref
Brazil, São Paulo	Leaves	HD	Dillapiole	Immersion	*Leishmania amazonensis* (Euglenozoa)	Leishmanicidal – growth inhibition of promastigote (IC_50_=59.4μm)	[73]
Brazil, Santa Catarina	Leaves	HD	(Z)-β-ocimene (7%), (E)-β-ocimene (13.9), safrole (6.2%), α-humulene (4.9%), α-humulene (20.9%), γ-cadinene (5.5%), spathulenol (5.3%)	Incubation	*Leishmania amazonensis* (Euglenozoa)	Leishmanicidal – Antipromastigote (IC_50_=25.9μg/mL) and antiamastigote (IC_50_=36.2μg/mL) activity	[7]
Cuba, La Habana	Leaves	HD	Piperitone (23.7%), camphor (17.1%), viridiflorol (14.5%)	Incubation	*Leishmania amazonensis* (Euglenozoa)	Leishmanicidal – Antipromastigote activity (IC_50_=23.8μg/mL)	[68]
Brazil, Minas Gerais (UFLA)	Leaves	HD	Nerolidol	Incubation	*Leishmania braziliensis* (Euglenozoa)	Leishmanicidal – Antipromastigote activity (IC_50_/24h=77.9μg/mL)	[105]
Brazil, São Paulo	Leaves	HD	Dillapiole	Immersion	*Leishmania braziliensis* (Euglenozoa)	Leishmanicidal – growth inhibition of promastigote (IC_50_=69.3μm)	[73]
Cuba, La Habana	Aerial parts	SD	NS	Incubation	*Leishmania braziliensis* (Euglenozoa)	Leishmanicidal – Antipromastigote activity (50.8μg/mL=100%)	[66]
–	–	–	*Dillapiole	Incubation	*Leishmania chagasi* (Euglenozoa)	Leishmanicidal – Antipromastigote activity (50μg/mL=99%)	[25]
Cuba, La Habana	Leaves	HD	Piperitone (23.7%), camphor (17.1%), viridiflorol (14.5%)	Incubation	*Leishmania donovani* (Euglenozoa)	Leishmanicidal – Antipromastigote activity (IC_50_=7.7μg/mL)	[68]
Cuba, La Habana	Leaves	HD	Piperitone (23.7%), camphor (17.1%), viridiflorol (14.5%)	Incubation	*Leishmania infantum* (Euglenozoa)	Leishmanicidal – Antiamastigote activity (IC_50_=8.1μg/mL)	[68]
Cuba, La Habana	Leaves	HD	Piperitone (23.7%), camphor (17.1%), viridiflorol (14.5%)	Incubation	*Trypanosoma brucei* (Euglenozoa)	Antitrypanosomal activity (IC_50_=2.0μg/mL)	[68]
Brazil, Minas Gerais (UFLA)	Leaves	HD	Linalool, nerolidol	Incubation	*Trypanosoma cruzi* (Euglenozoa)	Antitrypanosomal – activity against epimastigote (IC_50_/24h=84.7μg/mL at 28°C), amastigote (IC_50_/24h=9μg/mL at 37°C), cell-derived trypomastigote (IC_50_/24h=2.8 and 3.8μg/mL at 28°C and 4°C, respectively) and metacyclic trypomastigote (IC_50_/24h=12.1μg/mL at 28°C)	[15]
Cuba, La Habana	Leaves	HD	Piperitone (23.7%), camphor (17.1%), viridiflorol (14.5%)	Incubation	*Trypanosoma cruzi* (Euglenozoa)	Antitrypanosomal activity (IC_50_=2.1μg/mL)	[68]
Cuba, La Habana	Aerial parts	SD	NS	Incubation	*Trichomonas vaginalis* (Metamonada)	Trichomonacide (100μg/mL=100%)	[66]
Brazil, Ceará	Aerial parts (leaves and branches)	SD	Dillapiole (76.5%), piperitone (6.1%), terpinen-4-ol (2.3%), myristicin (2.1%), (E)-caryophyllene (1.5%), γ-terpinene (1.3%), germacrene-D (1.2%), apiole (1.2%)	Contact	*Plasmodium falciparum* (Apicomplexa)	Antiplasmodial activity (72h exposure, W2: 1.30ng/mL=100%; Dd2: 10.30mg/mL=77%)	[62]
Cuba, La Habana	Leaves	HD	Piperitone (23.7%), camphor (17.1%), viridiflorol (14.5%)	Incubation	*Plasmodium falciparum* (Apicomplexa)	Antiplasmodial activity (IC_50_=1.3μg/mL)	[68]
Brazil, Amazonas	Leaves	HD	dillapiole (76.2%)	Contact	*Haemonchus contortus* (Nematoda)	Egg-hatching inhibition (IC_50_=5.72mg/mL); Blocking larvae development (IC_50_=0.10mg/mL, IC_90_=0.34mg/mL)	[35]
Brazil, Minas Gerais	Leaves	HD	1,8-cineole (55.8%), α-terpineol (5.9%), trans-ocimene (4.8%), β-pinene (4.7%), α-pinene (4.5%), bicyclogermacrene (4.4%)	Contact	*Haemonchus contortus* (Nematoda)	Egg-hatching inhibition (LC_90_=8.9mg/mL)	[71]
Brazil, Amazonas	Leaves	SD	Dillapiole (92%)	Contact (ingestion of medicated food by fishes)	*Hysterothylacium* sp. (Nematoda)	Larvicide (64 mL/kg=76.21% at 15days treatment)	[18]

*Only the main compound was tested, Aerial parts: leaves and branches, HD: hydro-distillation, SD: steam-distillation, NS: not specified, EO: essential oil, LC_90_: lethal concentration for 90%, IC_50_: inhibitory concentration for 50%, IC_90_: inhibitory concentration for 90%.

When specified, the concentration of dillapiole was always higher than 75%. In this case, the EO was mostly applied through incubation in which it was added to the infected cell cultivation and placed in the best conditions (usually 37°C) for protozoan growth, for at least 24h. Efficiency was mostly expressed as activity against promastigotes. For instance, in the case of *Leishmania*, to inhibit the growth of at least 50% (IC_50_) of promastigotes, about 15μg/mL of the EO with dillapiole are needed as the main component.

Antiparasitic activities, mostly antileishmanial effects, represent the second largest group of results, and are supported by other studies showing these properties of various EOs, such as *Cryptocarya aschersoniana* EO [2] and others.

### Synergistic activity

Table 5 shows the activity of dillapiole in synergy with other chemical substances used as insecticides to determine potential efficacy against *Spodoptera frugiperda*, a caterpillar known as the fall armyworm, considered a pest due to the damage it causes to a wide variety of crops and the associated economic losses.

**Table 5:** Synergetic properties of dillapiole along with chemical insecticides.

Country	Part	Extraction	Main compound	Application	Insect	Activity	Ref
Brazil, Acre	Leaves	SD	dillapiole (71.9%)+(cypermethrin, zeta-cypermethrin, permethrin, esfenvarelate)	Topical and residual contact	*Spodoptera frugiperda* (Fall armyworm) (Lepidoptera)	Larvicide (topical: 0.54μL EO: +0.0553μL Cypermethrin LD_50_=0.0093μL/mg of insect; +0.000733μL Zeta-Cypermethrin LD_50_=0.00017μL/mg; +0.000327μL Permethrin LD_50_=0.000068μL/mg; +0.2μL Esfenvarelate LD_50_=0.053μL/mg)(residual: 584.9ppm EO: +256.70ppm Cypermethrin LC_50_=3.52ppm; +747.80ppm Zeta-Cypermethrin LC_50_=617.00ppm; +246.20ppm Permethrin LC_50_=14.30ppm; +48756.10ppm Esfenvarelate LC_50_=3640.70ppm)	[30]
Brazil, Acre	Leaves	SD	dillapiole (71.9%)+(α-cypermethrin, β-cypermethrin, fenpropathrin, γ-cyhalothrin)	Topical and residual contact	*Spodoptera frugiperda* (Fall armyworm) (Lepidoptera)	Larvicide (topical: 0.006μL EO: +0.0019μL α-Cypermethrin LD_50_=0.0000079μL/mg of insect; +0.015μL β-Cypermethrin LD_50_=0.0017μL/mg; +0.0022μL Fenpropathrin LD_50_=0.000064μL/mg; +0.0011μL γ-Cyhalothrin LD_50_=0.00011μL/mg) (residual: 0.000055μL EO: +0.0000016 α-Cypermethrin LD_50_=0.00000021μL/cm^2^; +0.0000015μL β-Cypermethrin LD_50_=0.0000016μL/cm^2^; +0.00000062 Fenpropathrin LD_50_=0.00000018μL/cm^2^; +0.00000019μL γ-Cyhalothrin LD_50_=0.000000033μL/cm^2^)	[29]
Brazil, Acre	Leaves	SD	dillapiole (71.9%)+(thiamethoxam/γ-cyhalothrin, γ-cyhalothrin, imidacloprid/β-cyfluthrin, β-cyfluthrin, teflubenzurom/α-cypermethrin, α-cypermethrin)	Topical and residual contact	*Spodoptera frugiperda* (Fall armyworm) (Lepidoptera)	Larvicide (topical: 0.000055μL EO: +0.000014μL Thiamethoxam/γ-Cyhalothrin LD_50_=0.0000016μL/insect; +0.00000038μL γ-Cyhalothrin LD_50_=0.000000065μL/insect; +0.00016μL Imidacloprid/β-Cyfluthrin LD_50_=0.000055μL/insect; +0.0000055μL β-Cyfluthrin LD_50_=0.000001μL/insect; +0.000011μL Teflubenzurom/α-Cypermethrin LD_50_=0.0000015μL/insect; +0.000012μL α-Cypermethrin LD_50_=0.0000016μL/insect) (residual: 584.85ppm EO: +183.4ppm Thiamethoxam/γ-Cyhalothrin LC_50_=74.1ppm; +1026.4ppm γ-Cyhalothrin LC_50_=11.7ppm; +8455.2ppm Imidacloprid/β-Cyfluthrin LC_50_=1512.6ppm; +927.3ppm β-Cyfluthrin LC_50_=16.0ppm; +1895ppm Teflubenzurom/α-Cypermethrin LC_50_=0.8ppm; +206.3ppm α-Cypermethrin LC_50_=10.1ppm)	[28]
Brazil, Acre	Leaves	SD	dillapiole (71.9%)+(profenofos, fenitrothione, chlorpyrifos, metomil)	Topical and residual contact	*Spodoptera frugiperda* (Fall armyworm) (Lepidoptera)	Larvicide (topical: 0.000055μL EO: +0.000038μL Profenofos LD_50_=0.0048μL/mg of insect; +0.00045μL Fenitrothione LD_50_=0.000071μL/mg; +0.000025μL Chlorpyrifos LD_50_=0.00001μL/mg; +0.0000067μL Metomil LD_50_=0.0000061μL/mg) (residual: 0.006μL OE: +0.0068 Profenofos μL LC_50_=0.0013μL/cm^2^; +0.0017μL Fenitrothione LC_50_=0.00044μL/cm^2^; +0.000053μL Chlorpyrifos LC_50_=0.000016μL/cm^2^; +0.0083μL Metomil LC_50_=0.0048μL/cm^2^)	[31]

SD: steam-distillation, EO: essential oil, LC₅₀: lethal concentration for 50%, LD₅₀: lethal dose for 50%.

In these experiments, two methods of application were used to determine the larvicidal effect: in the first case, they applied the solutions topically to the dorsal side of the larvae, whereas in the second, to verify the residual potential of the synergistic combination, they soaked filter paper in the substance, let it dry and placed it in a Petri dish where the larvae were then situated for 24h. The concentration of dillapiole was always 71.9% and one of the chemical compounds that was used by three of the four experiments was cypermethrin and its different forms. Larvicidal activity was systematically tested. These results reinforce the conclusions pointed out by various authors [5, 12] concerning synergistic activity.

## Conclusions

Clearly, the Amazon Rain Forest still harbours a huge amount of knowledge. Articles have described the proprieties of just some of the species from this region, and of these, thousands concerned EO properties.

In this review, the authors aim to increase interest in this useful species and its EO, and in a wider context, to highlight the possibility of finding natural substitutes for chemical biopesticides.

EOs are a promising natural alternative as insecticides, acaricides and antiparasitic products, but researchers and industry must address several challenges in order to obtain new commercial products. EOs are recognised as ecofriendly, biodegradable and cost effective raw materials [17] and generally present very low mammalian toxicity and short environmental persistence [80]. Unfortunately, precisely due to their short persistence, EOs exert a short duration of action and require several applications or additional efforts on formulation strategies. The most common formulation strategy is to add chemical fixatives or stabilizers to the EO, in order to prolong effects. Fixatives make it possible to slow evaporation of EOs due to their lower volatility in comparison with EOs compounds. Promising results with natural (vanillin) and synthetic fixatives (Glucam P-20, Fixolide) have been reported by Songkro *et al*. (2012) [98], with several repellent mosquito formulations. The presence of the fixatives was able to improve repellent activity and the longevity of formulation efficacy.

Concerning the mode of action, EOs are generally able to affect insects and mites through neurotoxic effects involving the inhibition of acetylcholinesterase and an effect on the octopamine synapses and GABA receptors. In particular, due to the presence of methylene dioxy rings, dillapiole may inhibit insect P450 cytochrome activity. This mechanism affects the phase I metabolism of xenobiotics, which is responsible for inactivating the insecticides. EOs also exert repellent activity, blocking the odour receptor neurons (ORNs) of the insects [17, 33, 80].

As reported by Misni *et al*. (2011) [63], a general approach to the understanding of the insecticidal mode of action of EOs should be similar to that for pyrethroids. After initial excitability, the insects lose the control of their movements and present convulsions, paralysis and ultimately, death.

Dillapiole seems to be the most valuable compound among those forming this EO and for the same reason should be extensively studied. Even though essential oils may be a source of allergy in humans, dillapiole has not been added to the list of 26 fragrance ingredients listed as allergens in Annex III of the European Union’s Scientific Committee on Consumer Safety Opinion on Fragrance allergens in cosmetic products (SCCS/1459/11). Interestingly, a study performed by Aciole *et al*. (2013) [1], on dillapiole identified genetic toxicity on somatic cells of *Drosophila melanogaster.* Hsuuw and Chan (2015) [44] investigated the effects of dillapiole on mouse oocyte maturation, showing a potential teratogenic effect. The authors suggest that the effect of dillapiole on human oocytes should also be investigated in order to assess the safety of its application as a drug or biocide. These findings indicate that dillapiole should be studied further to determine its possible toxicity in humans. For instance, it would be useful to evaluate its effects in synergy with other compounds to find the most useful match, as it has been shown that the oils are more effective when used in their totality than just in main component form. Other compounds are also of interest based on the data shown in the tables, as in some cases they are even the main compound instead of dillapiole, for example myristicin or 1,8-cineole. Therefore, a more specific study of these isolated components is required to have a wider understanding of the entire EO.

In addition, it is important to define the best way to investigate *P. aduncum* EO or dillapiole in open field studies or infested areas, in order to develop new phytoiatric formulations, such as nanoemulsions or insecticidal sugar baits, which can be successfully adopted as alternative commercial biopesticides. With the same approach, deeper pharmacological investigations that include formulation development should be performed in order to obtain new plant-based antiparasitic drugs.

We also need to understand the consequences of using *P. aduncum* EO as a biopesticide, such as its effect on the environment and its residual phytotoxicity, among others.

The proprieties and effects of this EO must be better analysed and clarified, as the results highlighted in this review are quite promising. Finally, further studies could focus on finding a semisynthetic product from this EO, which would be more economically practical and, at the same time, place lower demands on natural resources.

## Conflict of interest

The authors declare that there are no conflicts of interest.

## Author contribution

AD drafted the manuscript and revised the final version. MR participated in the data mining, the literature analysis, and manuscript editing. JBS and TRT provided additional information. TRT contributed to the conception of the research, monitored the study, and revised the final version. All authors reviewed and approved the final version of the manuscript.
